# Systematic standardization of organ-on-a-chip under controlled flow conditions: Use case with Caki-1 and A549 cell lines

**DOI:** 10.1016/j.mex.2026.104172

**Published:** 2026-09-22

**Authors:** Andrea Balsa-Díaz, Laura Vázquez-Vázquez, Elías Ferreiro-Vila, Lois Rivas-Meizoso, Antón L. Martínez, José Manuel Brea, Mabel Loza, Santiago Pérez Rodriguez, Esteban Sinde, Ezequiel Alvarez, Bruno K. Rodiño-Janeiro

**Affiliations:** a BFlow SL, Edificio Emprendia, Santiago de Compostela, Campus Vida, s/n, 15706, Spain; b Departamento de Farmacología, Farmacia y Tecnología Farmacéutica, Universidade de Santiago de Compostela (USC), Santiago de Compostela, 15782, Spain; c Drug Screening Platform/Biofarma Research Group, CIMUS Research Center, Universidade de Santiago de Compostela (USC), Santiago de Compostela, 15782, Spain; d Hifas Innovation Hub, Lugar de Portamiuño 7 Bora, Pontevedra, 36004, Spain; e Instituto de Investigación Sanitaria de Santiago de Compostela (IDIS), Complexo Hospitalario Universitario de Santiago de Compostela (CHUS). SERGAS, Travesía da Choupana s/n, A Coruña, Santiago de Compostela, 15706, Spain; f CIBERCV, Madrid, Spain

**Keywords:** Organ-on-a-chip, Microfluidic chip, 3D cell culture, Systematic standardization of microfluidic protocols

## Abstract

New *in vitro* models are urgently needed to improve the preclinical development of new drugs. Current standardised cellular models are mainly based on 2D cell culture, which lacks physiological flow conditions and often involves limited or simplified co-culture settings.

• Organ-on-a-chip (OoC) systems use microchips incorporating extracellular matrices, surface coatings and cell cultures to mimic physiological conditions, enabling the formation of 3D structures, co-culture systems and dynamic flow.

However, to perform reproducible and controlled experiments using OoC systems, it is necessary to systematically standardise cell culture conditions within the microfluidic channels.

• The selection of the extracellular matrix (ECM) coating of the chip must be tailored and optimised for each specific cell type used in OoC systems.

It is essential to establish the appropriate biological microenvironment following cell adhesion to the chip, including the time required for cell adhesion and maturation, as well as the appropriate flow conditions.

• This protocol standardises flow and ECM coating conditions in OoC with kidney (Caki-1) and lung (A549) cell lines, facilitating reproducible experiments and enabling broader application across different OoC models.

## Graphical abstract

**Figure:**
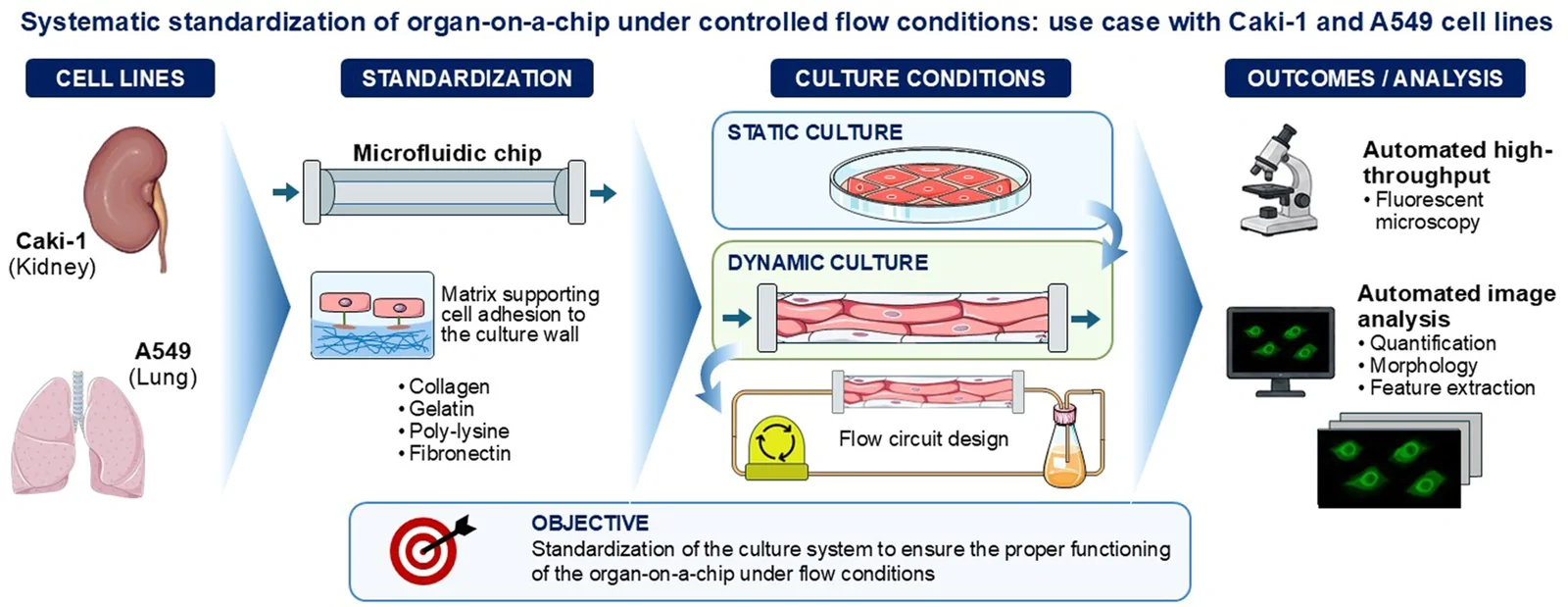

**Specifications table**

**Table:** 

**Subject area**	Pharmacology, Toxicology and Pharmaceutical Science
**More specific subject area**	Organ-on-a-chip models for biomedical research and drug discovery
**Name of your protocol**	Systematic standardization of cell culture for organ-on-a-chip under controlled flow conditions
**Reagents/tools**	REAGENTS: • A549 cell line (Elabscience, cat. no. CL-0016) • Caki-1 cell line (Elabscience, cat. no. CL-0052) • Collagen type I (indicated as collagen 1), rat tail 3 mg/mL (Gibco, cat. no. A1048301) • Collagen type I (indicated as collagen 2), rat tail 3–4 mg/mL (Corning, cat. no. 354236) • Gelatin from bovine skin (Sigma-Aldrich, cat. no. G9382) • Foetal bovine serum (FBS), Qualified (Gibco, cat. no. 10270-106) • Fibronectin bovine plasma 1 mg (Sigma-Aldrich, cat. no. F1141) • Ham’s F12 culture medium w/L-Glutamine (Biowest, cat. no. L0135–500) • Hoechst 93342 (NucBlueTM Live Cell Stain ReadyProbesTM Reagent, Invitrogen, cat. no. R37605) • Phosphate-buffered saline (PBS) tablets (Invitrogen, cat. no. 003002) • McCoy’s 5A culture medium w/L-Glutamine (VWR, cat. no. 392-0420) • Paraformaldehyde, 4% in PBS, ready-to-use fixative (Biotium, cat. no. 22023). !CAUTION: Paraformaldehyde is toxic. Use in fume hood and wear personal protection, such as gloves and goggles. • Penicillin-streptomycin solution 6,0/10,0 g/L 100X (P/S, Biowest, cat. no. L0022-100) • Poly-l-lysin solution 0.1% (w/v) in H_2_O (ChemCruz, cat. no. sc-286689) • Trypsin-EDTA 0.25% (w/v) (1X) (Gibco, cat. no. 25200-056) EQUIPMENT: • 3-stop platinum-cured silicone tubing 1 mm ID 3 mm OD (Darwin Microfluidics, SE-TUB-SIL-SSS-1 × 1) • Cell culture maintenance incubator (37 °C, 5% CO_2_, humidified; Eppendorf CellXpert) • Cell culture test incubator (37 °C, 5% CO_2_, humidified; Sanyo MCO-19AIC(UV)) • Inverted microscope (Olympus IX73P2F) • Male luer lock connector (BFlow, B02_0027) • Multi-linear channel chip - Set of 3 (BFlow, B06_0024) • Open jaw slide clamp (BFlow, B02_0018) • Reglo independent channel control (ICC) peristaltic pump (Ismatec, cat. no. MFLX78018-24-EU) • Reservoirs with septum cap (ThermoScientific, cat. no. 047422-4001) • Straight chip connector (BFlow, B02_0021) • Straight press-in plug (Set of 10, BFlow, B02_0029) • Syringe needle (BFlow, B02_0024) • Silicone tubing 1 mm ID 3 mm OD (BFlow, B02_0009) • Tubing connector (BFlow, B02_0005)
**Experimental design**	Human cell culture in microfluidic chips for organ-on-a-chip experiments allowing 3D vessel-like disposition and flow conditions.
**Trial registration**	Not applicable
**Ethics**	Not applicable. This protocol used cell lines available commercially.
**Value of the Protocol**	• Organ-on-a-chip (OoC) systems use microchips with extracellular matrices, coatings, and cell cultures to mimic physiological conditions, enabling 3D structures, co-culture, and dynamic flow. • The selection of the extracellular matrix (ECM) coating of polydimethylsiloxane chips for kidney (Caki-1) and lung (A549) cell lines culture was optimized. • This protocol standardizes flow conditions in OoC with kidney (Caki-1) and lung (A549) cell lines, facilitating reproducible experiments and broader application across OoC models.

## Background

New *in vitro* models are urgently needed to improve both the quality of research data and the preclinical development of new drugs. Multiwell 2D cell culture models have been widely used for almost 100 years [1], however, these culture conditions are not optimal for obtaining reliable and physiologically relevant biological data. More recently, alternatives to these traditional culture systems have been developed, including advanced 3D cellular models such as organoids, spheroids and organ-on-a-chip (OoC) systems. Among these models, OoC technology has emerged as a revolutionary approach in biomedical research. These sophisticated 3D models mimic physiological tissular architecture and enable the generation of customised cellular microenvironments with precise fluidic and structural control [[2], [3], [4]].

These models have emerged as an improvement over both conventional 2D cell culture [5] and animal models, which, although widely used, often generate results that cannot be directly translated to humans [[6], [7], [8]]. Compared with conventional 2D cell models, OoC systems exhibit differences in biological behaviour, including cellular migration and proliferation [9,10], cell-cycle regulation and metabolic pathways [10] and, importantly, a more physiologically relevant response to treatments [11,12]. Importantly, the characteristics and underlying concept of OoC technology may contribute to reducing and partially replacing the use of animals in research. In particular, these models may help address interspecies differences and ethical concerns associated with animal experimentation [13]. The combination of the advantages of *in vitro* models with increased physiological complexity [14] has generated considerable interest in the application of OoC systems as preclinical *in vitro* models. These systems provide a highly controlled cellular environment, good reproducibility and relatively straightforward assembly.

Flow within the chip is a relevant factor because it reproduces wall shear stress conditions [15], which can elicit biological responses affecting cell differentiation, morphology and metabolism [16], thereby providing a closer representation of the physiological environment [17]. The type and the pattern of flow depend on both the geometry of the chip and the external perfusion system, resulting in different flow profiles and, consequently, distinct cellular responses [18].

Another relevant factor mimicking the physiological microenvironment and enabling cells to withstand flow is the nature of the cell-adhesion surface. Polydimethylsiloxane (PDMS) is a widely used material for OoC fabrication owing to its attractive properties, including biocompatibility, gas permeability and favourable mechanical and optical properties [19]. However, PDMS is inherently hydrophobic, which results in poor cell-adhesion capacity. Consequently, biological functionalisation plays a central role in OoC development, with extracellular matrix (ECM) coatings being one of the most widely used strategies to improve surface hydrophilicity [20] and provide chemical (such as hydroxyl, amine or carboxyl groups) and biological cues (such as peptide sequences) that promote cell attachment. Different ECM coatings can influence cellular responses [21], resulting in differences in cell adhesion, biomarker production, growth rate, morphology and tight-junction protein expression [22,23].

As OoC systems offer promising applications in drug research, particularly for evaluating the efficacy and toxicity of compounds, the development of reliable and accurate *in vitro* models is essential. Characterisation of both flow conditions and optimised ECM coatings in newly developed OoC systems will enable the generation of high-quality, reproducible models of human organs and diseases. In this work, we experimentally standardised flow parameters and ECM coating conditions using two cell lines derived from lung (A549) and kidney (Caki-1) tissues. This approach enabled us to establish optimised conditions to ensure the reliability and consistency of OoC models.

## Materials

### Reagent setup

**Phosphate-buffered saline (PBS)**: dissolve each tablet of PBS 100 mL of distilled water. PBS solution was autoclaved at 121 °C for 20 min for sterilization.

**Gelatin solution (0.02% and 0.2% w/v):** weight out the appropriate amount of gelatin for each percentage solution and dissolve in distilled water (for example 20 mg in 100 mL of distilled water for 0.02% w/v). The solution is autoclaved at 121 °C for 20 min, allowing the complete solubilization.

**Gelatin solution (0.02% and 0.2% w/v) with fibronectin 5** **μg/mL:** add the appropriate amount of fibronectin to obtain 5 μg/mL in gelatin solution 0.02% in distilled water and proceed the same way with gelatin solution 0.2%.

**Collagen 100** **μg/mL in water or PBS:** add the appropriate amount of collagen to obtain 100 μg/mL in distilled water (collagen in water) or 1X PBS (collagen in PBS).

**Collagen 100** **μg/mL in water with fibronectin 5** **μg/mL:** add the appropriate amount of fibronectin to achieve 5 µg/mL in collagen 100 µg/mL in distilled water.

**Poly-l-lysin Solution 0.1% (w/v) in water:** the solution is not sterile, and it must be filtered with a 0.22 µm pore filter before use.

**Staining solution:** prepare a solution in serum-free medium with NucBlue 2 drops/mL.

*Note: collagen solutions prepared in this protocol were used for channel functionalization, not for hydrogel preparation.*

## Procedure

In this example, multi-linear channel chips have been used, but the same approach can be used in other OoC systems. Global timing of the protocol is represented in Fig. 1.

**Fig. 1:**
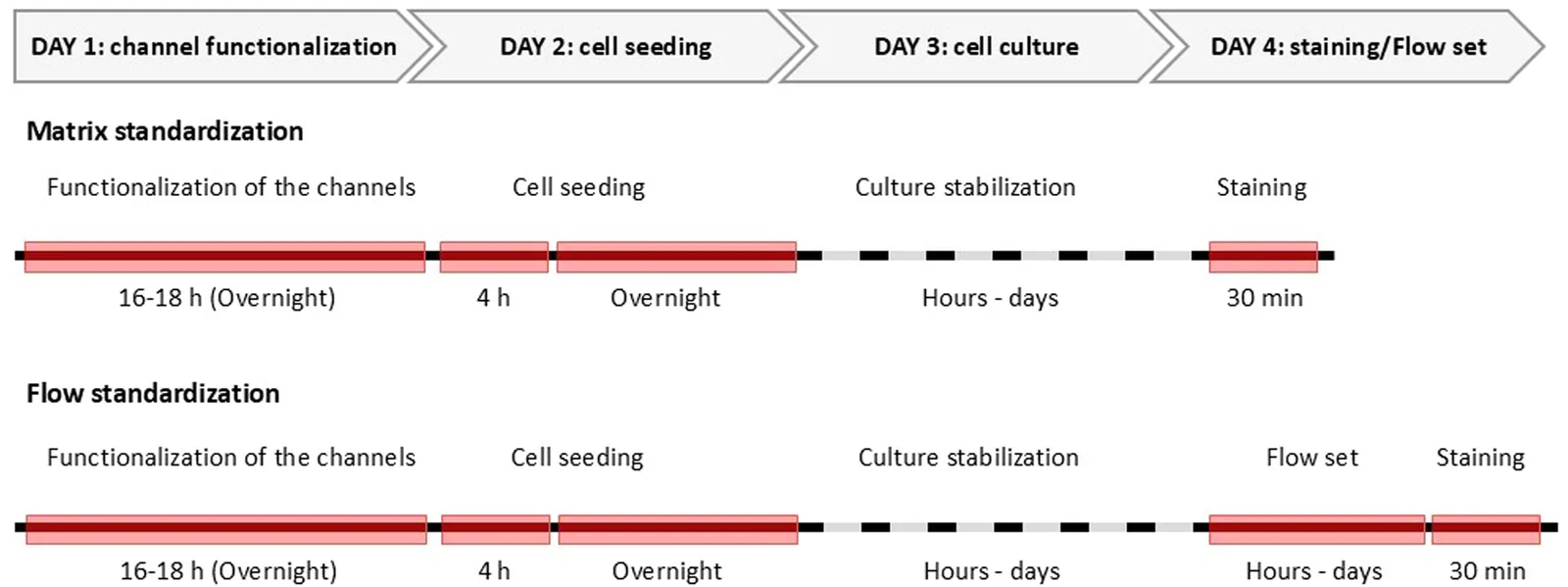
Schematic representation of the timing for both experimental settings, matrix and flow standardizations.

### Cell maintenance

A549 and Caki-1 cell lines were used to perform both matrix and flow standardization. All culture media were supplemented with foetal bovine serum (FBS, 10%) and penicillin-streptomycin (P/S, 1%). Trypsinization has been inactivated using complete medium in all cell lines. A549 cells were passaged at confluence using 0.25% (w/v) trypsin-EDTA for 2 min, recovered by centrifugation at 300 g for 3 min, split at a 1 plate to 4 ratio and maintained in culture in Ham's F12 medium w/L-Glutamine (146 mg/L) with 10% (v/v) FBS and 1% P/S. Caki-1 cells were passaged at confluence using 0.25% trypsin-EDTA for 2 min, recovered by centrifuging at 300 g for 3 min, split in a 1 plate to 6 ratio and maintained in culture in McCoy's 5A w/L-Glutamine (146 mg/L) with 10% FBS and 1% P/S.

### Matrix standardization

#### Functionalization procedure

1. Prepare the coating solutions to be tested (described at REAGENT SETUP section). The following coatings were used: Gelatin solution at 0.02% and 0.2% (w/v); Gelatin 0.02% and 0.2% with fibronectin 5 μg/mL; Collagen 100 μg/mL; Collagen 100 μg/mL with fibronectin 5 μg/mL and Poly-l-lysin solution 0.1% (w/v).
2. Place the autoclaved chip in a petri dish to improve handling.
3. Wash the channels with 1X PBS twice, before functionalization. *SEE TROUBLESHOOTING*
4. In each channel, place the desired coating solution by pipetting. Each channel of multi-linear channel chips (BFlow, B06_0024) holds approximately 80 µL.
5. Cover the inlet and outlets with straight press-in plug and incubate in the cell culture maintenance incubator for 16–18 h or overnight.
6. *PAUSE POINT:* The functionalized chip can be stored in the fridge for a couple of days. It is important to seal the petri dish with parafilm to prevent dehydration. Additionally, you can place small reservoirs with water or PBS on the plate further prevent dehydration.

#### Cell seeding

1. Confluent cells are trypsinised and counted using a Neubauer chamber.
2. The required cell concentration for chip seeding is 1.5·10^6^ cells per millilitre.
3. Remove the chip from the maintenance incubator and take out the straight press-in plug from the channels. *SEE TROUBLESHOOTING*
4. Remove the coating solutions from the channels.
5. Fill the channels with the cell suspension prepared previously.
6. Place straight press-in plugs in the inlet and in each outlet. Remove the overflowed medium from the channels when plugs are inserted.
7. Incubate upside down for 4 h to allow the cell to cover the top part of the channel.
8. After 4 h, take out straight press-in plugs and repeat the seeding process: fill the channels with the cell suspension and place the plugs, removing the excess of medium.
9. Incubate the channels face up for 16–18 h (overnight), to allow the cells to cover the bottom part of the channel.
10. Incubate the cells as long as required according to the needs of the experiment, the medium should be renewed daily.

#### Live cell staining

1. After the incubation of cells for the selected experimental time, remove the chip from the maintenance incubator and take out the straight press-in plug from the channels.
2. Add the staining solution to each channel and incubate for 30 min at room temperature and in the dark.
3. After 30 min remove the staining solution, replace with normal medium and place straight press-in plugs in the inlet and in each outlet.

#### Image acquisition

Cell number was quantified after staining using an Operetta CLS High Content Analysis System (Revvity, serial number 1600L21508). Both image acquisition and analysis were fully automated using Harmony v4.9.2137.273 software. For image analysis, an algorithm was developed to collect raw images without brightfield correction and to integrate the 50 planes acquired for each field into a single image. Subsequently, nuclei were identified using the predefined B protocol available in the software, which is suitable for analysing images containing relatively well-separated nuclei, although some background may be present. The area and roundness of the detected cells were then determined, and artefacts were excluded based on these parameters. Finally, the software was configured to report the number of nuclei classified as valid, excluding detected artefacts). Fourteen fields were selected to cover the entire length of the microfluidic channels.

For A549 cells, 50 planes separated by 5 µm were acquired, starting at a height of 500 µm and extending to 795) µm. For the Caki-1 cell line, 50 planes separated by 5 µm were acquired, starting at a height of 600 µm and extending to 895. A total of 14 fields were captured using a 5x objective, generating images with a resolution of 1080px X 1080px. The pixel resolution was 2.39 µm per pixel, corresponding to a field of view of approximately 2581 × 2581 µm.

All fields of view from the same experiment were merged using ImageJ (v1.54f) to determine the nuclei count for each technical replicate. Between four and seven technical replicates were performed for each experiment, and the mean value was used as the final result. At least three independent experiments were conducted for each experimental condition. Images were acquired under the conditions described in Table 1. Following image acquisition, cells were fixed according to the procedure described in Appendix 1.

**Table 1:** Fluorescence conditions for image acquisition.

Channel	Ex/Em (nm)	Exposure time (ms)	Intensity (from maximum)
Hoechst 3342 channel (NucBlue)	355–385/430–500	10	20%
Brightfield	-/-	1	20%

### Flow standardization

#### Functionalization of the channels

1. Coating matrixes selected from the best results from “**Matrix standardization**“ were applied in the flow standardization.
2. Prepare the desired coating matrixes (described in the reagent setup). For the functionalization of a single chip, 500 µL of the matrix solution must be enough.
3. For the functionalization of the channels, follow steps of the “Functionalization procedure” described above.

#### Cell seeding

To culture the cells, follow the steps described in the “Cell seeding” protocol.

#### Flow set up

1. All the elements used for the assembly of the flow (Fig. 2A, B) must have been previously autoclaved and all montage must be performed in a laminar flow hood under sterile conditions.

**Fig. 2:**
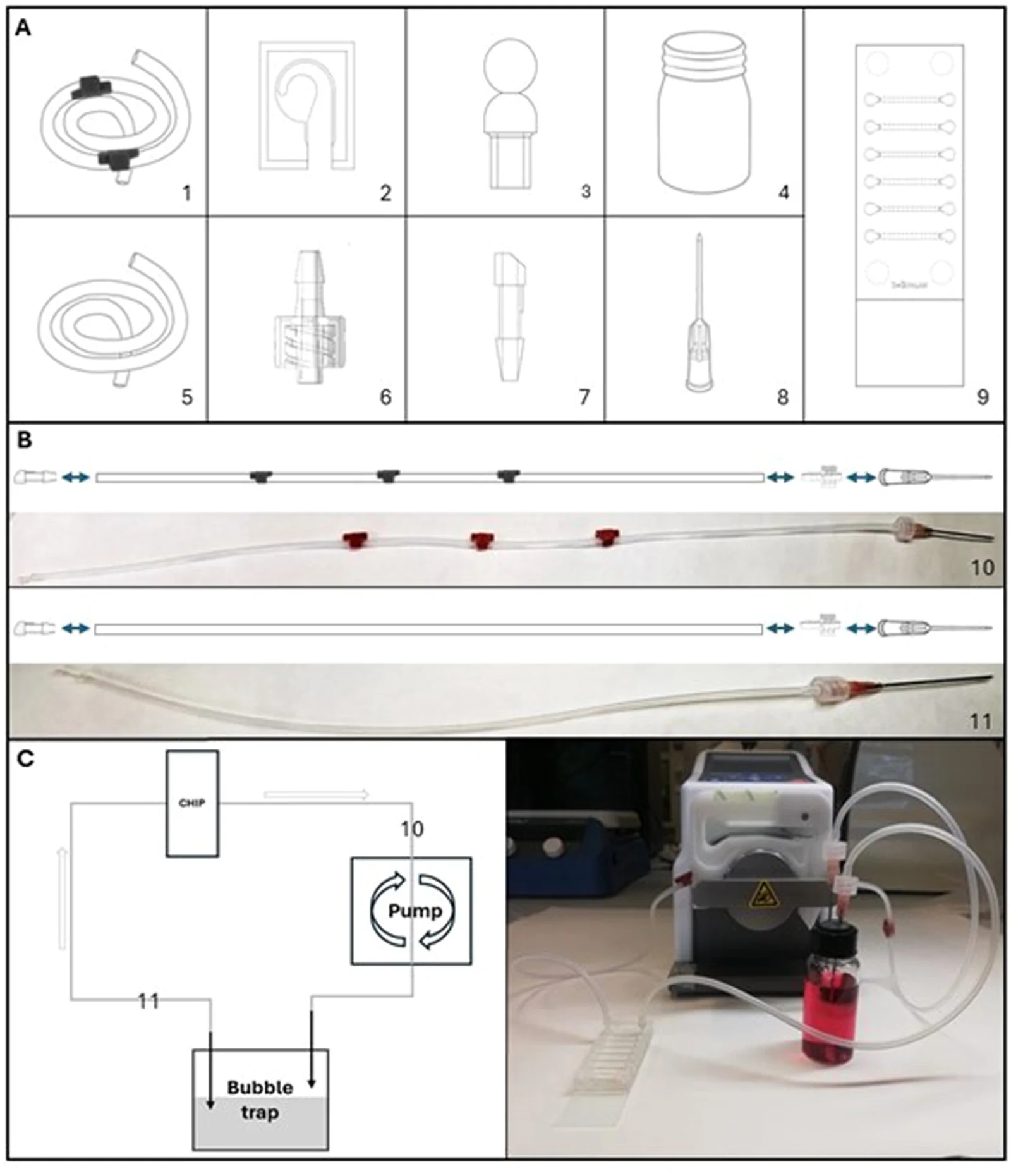
Schematic representation of the materials to set the experiment under flow. A) The individual materials are numbered as follows: 1–3-stop platinum-cured silicone tubing, 2-Open jaw slide clamp, 3-Straight press-in plug, 4-Reservoirs with septum cap, 5-Silicone tubing, 6-Male luer lock connector, 7-Straight chip connector, 8-Syringe needle, 9-Multi-linear channel chip. B: Representation of the assembly of the tubes, divided in two settings. 10-Tube from chip through the pump and to the reservoir composed by the materials 7 + 1 + 6 + 8 indicated in Fig. 2A; 11-Tube from reservoir to the inlet composed by the materials 7 + 5 + 6 + 8 indicated in Fig. 2A. C: Schematic representation of the system on the left (including the chip (material 9), the tube connecting the outlet of the chip and the reservoir through the pump (material 10), the bubble trap with the reservoir (material 4) and the tube from the reservoir to the inlet of the chip (material 11), the real mounted system is showed on the right.
2. For each flow channel, two tubes must be fitted with a male luer lock connector and a syringe needle at one end and a straight chip connector at the other end (Fig. 2B). One of those tubes must be a tube with stoppers to be placed in the peristaltic pump (Fig. 2B). For convenience, this step can be carried out before sterilisation.
3. Place medium, previously warmed, in each of the reservoirs. You must use one vial for each flow channel.
4. Place the syringe needle end of both tubes through the septum cap of the reservoir, one inside of the medium and the other not reaching the medium (placed in the air chamber of the half full reservoir). This set-up is specified in Appendix 2 and it will work as a bubble trap. *!CAUTION The straight chip connector and the ends of the tubes must be kept sterile*.
5. Place the tubing with the stoppers in the peristaltic pump cassette.
6. The tube that goes from the vial to the chip must be primed. Activate the pump and let the medium flow through, when the medium starts to come out of the end of the tube, stop the pump. Then place an Open Jaw Slide Clamp near the connector end of the tube. Repeat this step with all the tubes to be used into the inlet.
7. When all the tubes are in place and primed, the chip can be connected. Remove the plugs from the first channel and connect the straight chip connector end of the tube, which comes from the vial to the first channel inlet. Then, connect the other straight chip connector end of the tube, which goes to the pump, to the outlet (Fig. 2C). The process is repeated for each channel in the chip.
8. Once all the channels are connected to the tubes, remove the open jaw slide clamps.
9. Turn on the pump and check that the flow is properly established, without bubbles or leaks in the circuit. *SEE TROUBLESHOOTING*
10. Move the set up to the cell culture test incubator to keep the cells at the appropriate temperature. Leaves the flow running as long as necessary for experimental purposes.

#### Live cell staining

1. Remove the set up with the chip from the test incubator and take out the tubes from the channels.
2. For the cell staining, follow the steps described in “Live cell staining” protocol.

#### Image acquisition

For image acquisition, follow the indications in the “Image acquisition” protocol above.

### Troubleshooting

List of troubleshooting is presented in Table 2.

**Table 2:** Troubleshooting table.

Step	Problem	Possible reason	Solution
Functionalization procedure	When trying to fill the channel, the liquid does not enter the channel (flowing out).	Loose fit of the pipette tip into the chip inlet.	The tip of the pipette should tightly fit the canal inlet, if it is loose, then the tip should be cut to fit tighter.
Cell seeding	When the plugs are removed, bubbles are generated in the channel.	The plugs are removed very harshly	Remove the plugs by turning them around so that the air enters slowly and does not bubble up in the channel.
Flow set up	The flow rate is lower than expected or marked by the pumping system	There are leaks in the circuit	Revise possible leaks in the chip. Special attention should be taken with the tube with stoppers, since they are more prone to be broken/cracked
Flow set up	Abnormal production of bubbles after passing by the pumping system	The circuit is broken in the pumping system	Replace the tube with stoppers.

## Protocol validation

The described protocol was applied using the indicated multi-linear channel chips (BFlow, B06_0024) with two cell lines, Caki-1 and A549, being relevant cell cultures to test toxicity in kidney and lung, respectively. For the matrix standardization, ten different matrixes were tested. Matrixes that worked in static conditions were selected to be tested under flow conditions.

### Matrix standardization of Caki-1 and A549 cell lines

A total of three biological replicates has been made for each of the matrix conditions for each cell line, analysing the number of cells using the nuclear staining in Caki-1 and A549 (Fig. 3, Fig. 4, respectively).

**Fig. 3:**
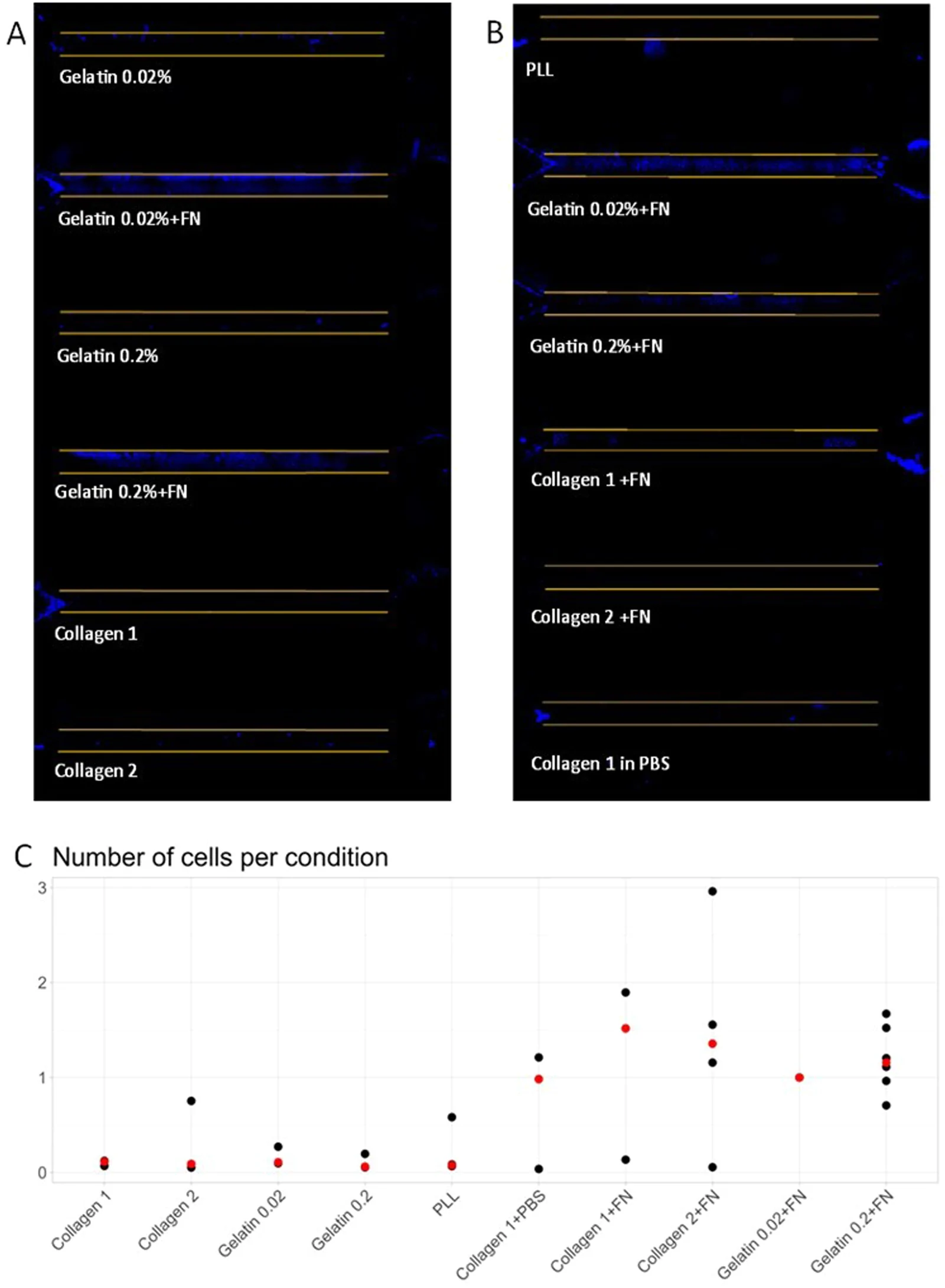
Caki-1 cell line matrix standardization. A) and B) representative fluorescent microscopy of the multi-linear channel chips showing the stained cell nuclei in blue for all the tested conditions. Yellow lines are showing the edges of the channels, being 1 mm the distance between lines. C) Dot plot illustrating the effectiveness of the ten types of matrixes assessed. Relative values with gelatine 0.02% + fibronectin are showed. The red dot represents the median value. (For interpretation of the references to color in this figure legend, the reader is referred to the web version of this article.).

**Fig. 4:**
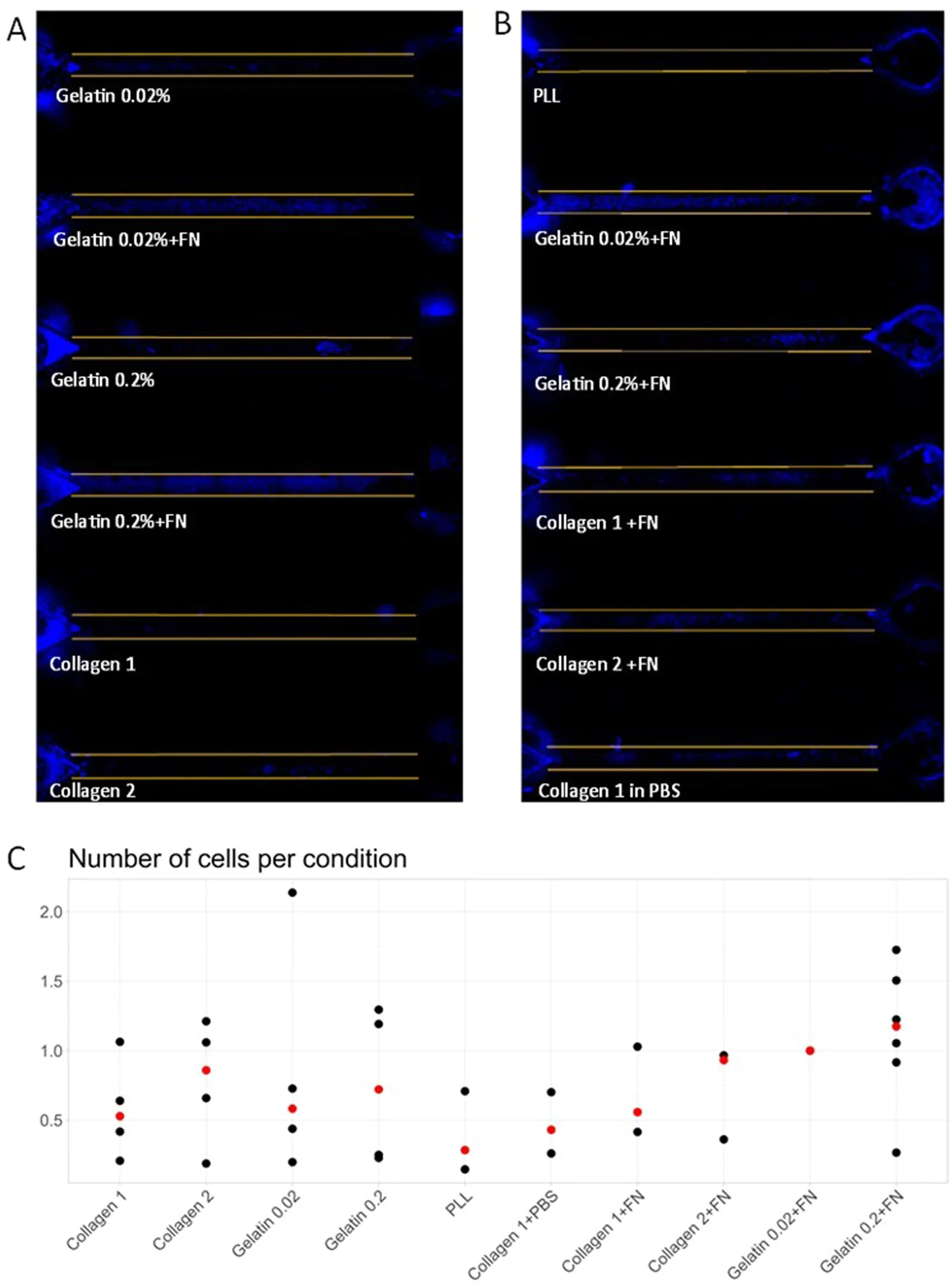
A549 cell line matrix standardization. A and B) representative fluorescent microscopy images of the multi-linear channel chips showing the stained cell nuclei in blue for all the tested conditions. Yellow lines are showing the edges of the channels, being 1 mm the distance between lines. C) Dot plot illustrating the effectiveness of the ten types of matrixes assessed. Relative values with gelatine 0.02% + fibronectin are showed. The red dot represents the median value. (For interpretation of the references to color in this figure legend, the reader is referred to the web version of this article.).

In Caki-1 cell culture, coating with the combination of gelatine and fibronectin (at both concentrations of gelatine) showed higher number of cells than gelatine alone (Fig. 3). The combination of collagen and fibronectin (using both collagens) showed lower adhesion of cells compared with gelatin+fibronectin coating. As in the case of gelatine, the combination of collagen and fibronectin improve the adhesion compared with collagen alone. Poly-l-lysine (PLL) functionalization did not show Caki-1 cell adhesion (Fig. 3). Relative values of the number of cells are represented in Fig. 3C. Performing a Mann-Whitney U test comparing the number of nuclei in gelatine 0.02%+fibronectin and each of the other groups, showed that collagen (1, from Gibco, and 2, from Corning), gelatine 0.02%, gelatine 0.2% and PLL were significantly different.

In A549 cell culture, no clear differences were observed. Gelatin alone provided low and no reproducible adhesion (Fig. 4). However, when fibronectin was added, 60% the experiments exhibited A549 cell adhesion (Fig. 4). In the case of collagen coating, A549 cells showed poor adhesion with collagen alone (same results with collagen 1 and 2), but upon addition of fibronectin, 50% of the experiments exhibited cell adhesion albeit with a uniform distribution. Consistent with the findings with Caki-1 cells, it appears that PLL functionalization did not exhibit cell adhesion (Fig. 4). No significant effect was observed using the Mann-Whitney U test compared to the Gelatine 0.02%+fibronectin group. These observations underscore the importance of matrix selection and standardization for microenvironment improvement, particularly the inclusion of fibronectin, promoting cell adhesion for both Caki-1 and A549 cells.

### Flow standardization of Caki-1 and A549 cell lines

Based on the analysis of the previous results, the most effective combination for channel functionalisation under flow conditions was identified as gelatine plus fibronectin for both cell types. The ability of cells cultured in an OoC system to withstand flow is directly related to the quality of cell adhesion and the establishment of an appropriate biological microenvironment. Therefore, it is important to determine the critical flow levels that these cell lines can withstand in order to establish the upper limit of wall shear stress suitable for OoC experiments. To achieve effective flow standardisation of flow conditions in organ chips, seven different flow conditions were investigated, covering a wide range of flow rates from 160 to 5000 µL/min (Table 3), corresponding to wall shear stress values ranging from 0.40 to 12.65 dyne/cm^2^ (Table 3). Wall shear stress was calculated using the Hagen–Poiseuille equation for a circular channel, τ = 4μQ/πR³, where µ is dynamic viscosity, Q is the volumetric flow rate and π is the constant. It was assumed a Newtonian fluid, a laminar flow, and that the flow characteristics do not change along the length of the channel.

**Table 3:** Flow rate and the corresponding wall shear stress in the channels used (1 mm of diameter with circular section). The referenced images are from Fig. 5, Fig. 6.

Image	Flow rate	Wall shear stress τ
**A**	160 µL/min	0.40 dyne/cm^2^
**B**	320 µL/min	0.81 dyne/cm^2^
**C**	640 µL/min	1.62 dyne/cm^2^
**D**	1200 µL/min	3.04 dyne/cm^2^
**E**	2500 µL/min	6.32 dyne/cm^2^
**F**	5000 µL/min	12.65 dyne/cm^2^
**G**	0 µL/min	0 dyne/cm^2^
**H**	STATIC	-

A comprehensive evaluation of the flow conditions provided significant insights into the behaviour of both cell lines in response to variations in flow. After flow exposure, cells were imaged as previously described to assess monolayer confluency. Monolayers with >95% surface coverage were considered to have intact cellular adhesion. Under all tested conditions, both Caki-1 (Fig. 5) and A549 (Fig. 6) cell lines remained adhered to the channels walls, suggesting that this microfluidic system is robust in maintaining cellular adhesion and integrity across a broad range of flow conditions. Qualitative assessment of the fluorescence images revealed alterations in Caki-1 nuclear morphology under flow conditions. Specifically, a non-quantifiable trend toward increased nuclear rounding was observed in the presence of flow (data not shown), suggesting a potential adaptive response to fluid-induced shear stress.

**Fig. 5:**
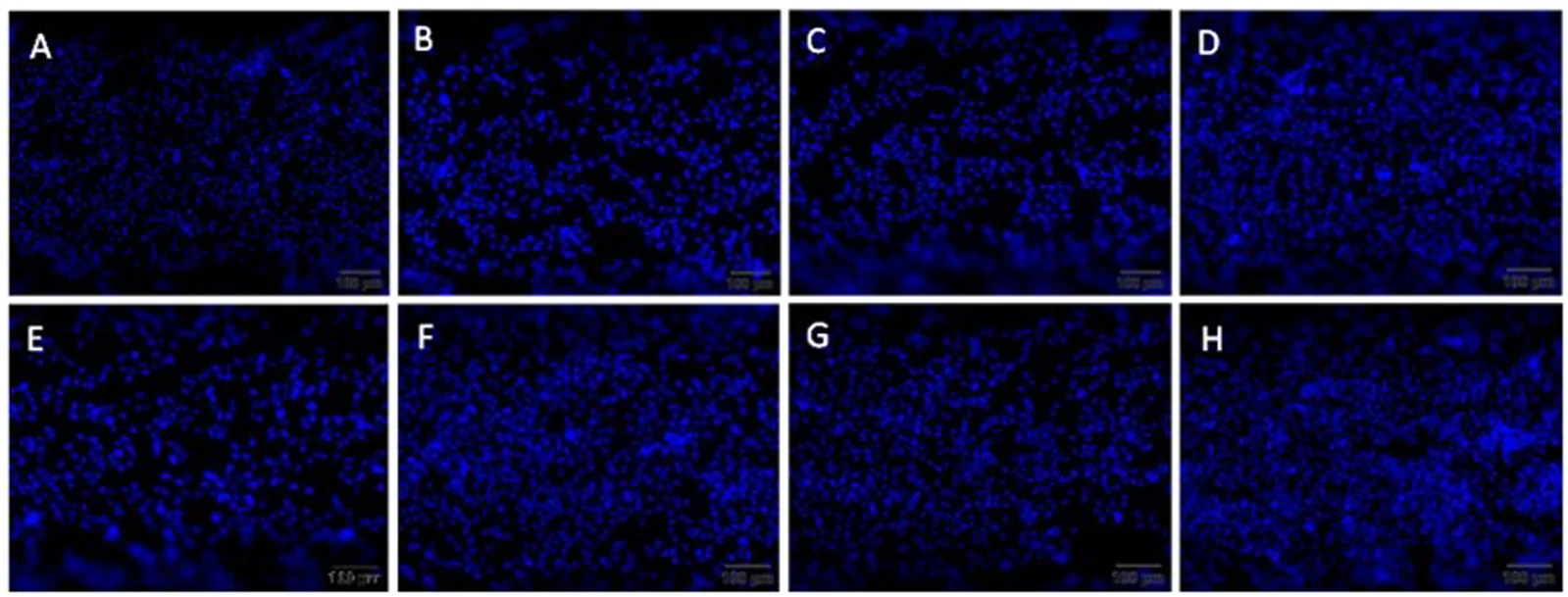
Caki-1 cell line under flow. Representative fluorescent microscopy images of the multi-linear channel chips showing the stained cell nuclei in blue for all the tested conditions in µL/min: A) 160, B) 320, C) 640, D) 1200, E) 2500, F) 5000, G) 0, H) static (as indicated in Table 3). The scale bar is 100 µm. (For interpretation of the references to color in this figure legend, the reader is referred to the web version of this article.).

**Fig. 6:**
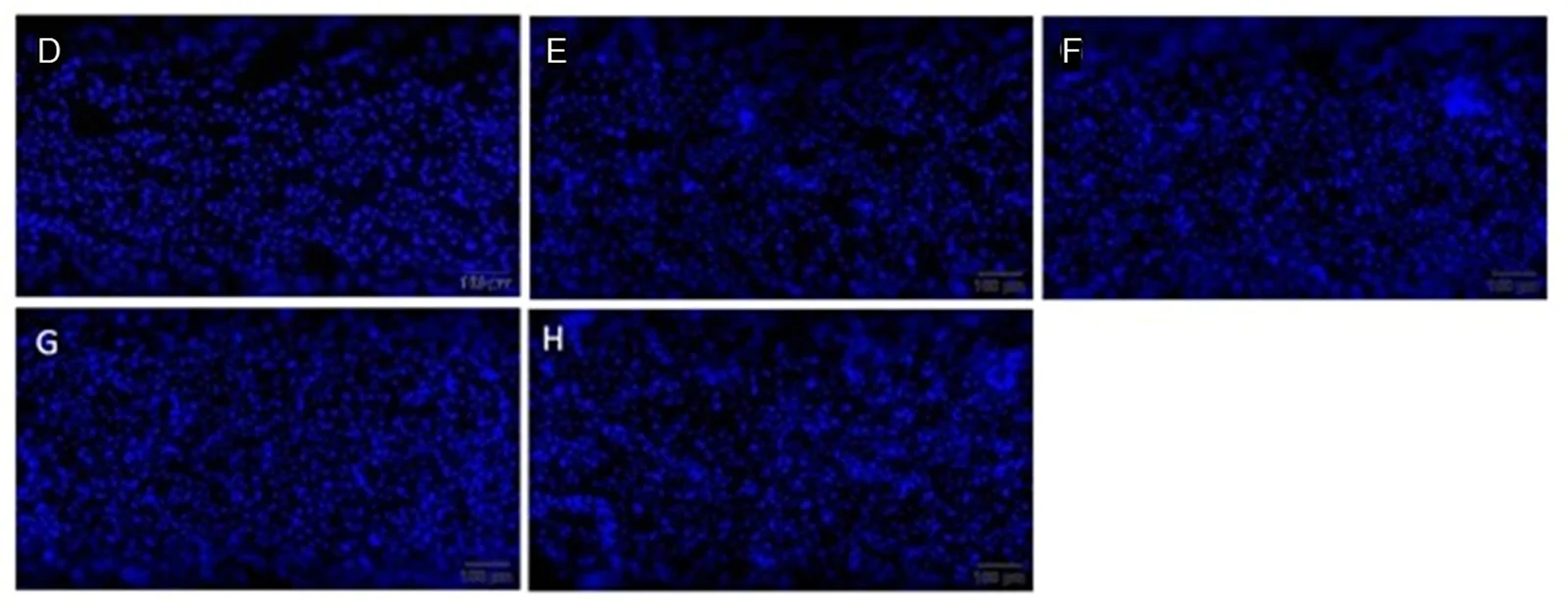
A549 cell under flow. Representative fluorescent microscopy images of the multi-linear channel chips showing the stained cell nuclei in blue for all the tested conditions in µL/min: D) 1200, E) 2500, F) 5000, G) 0, H) static (as indicated in Table 3). The scale bar is 100 µm. (For interpretation of the references to color in this figure legend, the reader is referred to the web version of this article.).

These findings not only demonstrate the ability of both cell types to withstand a broad range of flow conditions but also provide valuable insights into how the microfluidic environment may modulate cellular morphology and behaviour. The protocol described in this study aims to guide OoC users in the rational standardisation of flow conditions and in the identification of the operational limits of new OoC systems. This systematic approach provides insights into how different flow rates can influence cellular responses and system dynamics and may be applicable to other combinations of microfluidic channels, cell cultures and ECM coatings.

### Final considerations

In summary, three main considerations should be addressed when implementing experimental procedures in OoC systems under flow conditions.

First, the selection of the ECM or surface coating of the chip must be tailored and optimised for each specific cell type. Different cell types exhibit varying adhesion capacities, and not all materials or surface coatings are suitable for cell culture. As cells in these systems are exposed to wall shear stress generated by fluid flow, it is crucial to ensure robust cell adhesion and its maintenance throughout the experiment. As demonstrated in this study, the incorporation of fibronectin into the surface coating enhanced cell adhesion in both the Caki-1 and A549 cell lines.

Second, it is essential to establish an appropriate biological microenvironment following cell adhesion to the chip. This can be achieved by optimising several parameters, including allowing sufficient time for cell adaptation and maturation within the chip, using an appropriate culture medium, and applying a low flow rate before exposing the cells to the final experimental conditions. These steps are essential to ensure optimal cellular performance during subsequent experimental procedures.

Finally, the aim of OoC experiments is to analyse cellular behaviour and responses within a novel microenvironment under flow conditions. This requires the measurement and consideration of additional parameters to accurately assess cell functionality. Furthermore, it is important to recognise that these experiments may provide new insights into cellular responses that have previously been characterised under static culture conditions.

## Limitations

This protocol has been developed for microfluidic chips made of PDMS. Therefore, the matrix standardization described is only applicable to this material. For other materials a new protocol should be adapted. In the same way, the OoC application to other type of cells would obligate to an adaptation of the conditions of ECM and flow.

## Declaration of interest

The authors declare the following financial interests/personal relationships which may be considered as potential competing interests:

Andrea Balsa-Díaz, Laura Vázquez-Vázquez, Elías Ferreiro-Vila, Lois Rivas-Meizoso and Bruno Kotska Rodiño Janeiro were employees of BFlow SL during the collection and analysis of the results. Ezequiel Álvarez is co-founder of BFlow SL.

## Data Availability

Data will be made available on request.
