# Supplementary material for: Systematic standardization of organ-on-a-chip under controlled flow conditions: Use case with Caki-1 and A549 cell lines
Source: MethodsX. 2026 Sep 22;17:104172. doi: 10.1016/j.mex.2026.104172 (PMC13636011; doi:10.1016/j.mex.2026.104172)
Supplement: Supplementary file 1 [file mmc1.docx]

**Supplementary material *and/or* additional information**

**Appendix 1**

**Appendix 1 |** Fixation

1.	After the image acquisition step, the cells can be fixated inside the channel.

2.	Take out the Straight Press-in plug from the channels and remove the medium.

3.	Wash the channels with 1X PBS twice.

4.	Add paraformaldehyde fixation solution and incubate for 20 minutes. *CAUTION!: Paraformaldehyde is toxic. Use a fume hood and wear personal protection, such as gloves and goggles.*

5.	After 20 minutes remove the fixation solution and wash the channels with 1X PBS twice.

6.	Fill the channels with 1X PBS and cover them with Straight Press-in plug.

7.	Now you can store your chips until the analysis. The chips must be protected from light to avoid fluorescence bleaching.

*Note: it is important to note that viability stains will be lost with fixation.*

**Appendix 2**

**Appendix 2 |** Bubble trap

The purpose of this mechanism is to prevent bubbles from entering the microfluidic chip and disturbing the flow as well as the cells. For the bubble trap to function properly, the needle connected to the tube with stoppers coming from the pump must not be into the medium inside the reservoir, allowing the medium to drip and the needle connected to the tubing going into the chip must be submerged inside the medium to prevent air from entering into the chip.
